# The Practitioner and the Professional Soldier: Their Relations in the R.A.M.C.

**Published:** 1915-11-06

**Authors:** 


### THE PRACTITIONER AND THE PROFESSIONAL SOLDIER.

### THEIR RELATIONS IN THE R.A.M.C.

The present condition of the commissioned cadres of the Royal Army Medical Corps is that for every Regular Army officer of the Corps serving abroad there are at least ten temporarily commissioned who are in normal times engaged in civilian practice of one kind or another. This is, of course, well known and is a state of affairs which was deliberately contemplated long before the war by those who planned the organisation of the Royal Army Medical Corps and the scheme of augmenting its *personnel* in war-time. That the system works well is sufficiently attested by the reputation which the R.A.M.C. has won during the war. It is justifiably credited to the Corps by disinterested observers that never in history has a British Army been so well served by its Medical Service; and it would not be exaggerating to say that no branch of the Service has surpassed the Corps in technical efficiency. That is not to say that the Army Medical Service is perfect: grumbles on minor points have been heard from various quarters, and some at least of them have a certain amount of foundation. But, broadly speaking, the Medical Service does deserve the compliments which have been freely bestowed upon it; and the way in which its organisation has stood the searching tests of this titanic struggle is evidence that the lines on which it was laid out (mainly by Sir A. Keogh when he was Director-General some years ago) were sound and practical.

### The Expansion of the R.A.M.C.

The principle of this organisation is, as has been indicated, the absorption into the R.A.M.C. of enormous numbers of civil practitioners, temporarily commissioned and Special Reservists for the most part. Since this absorption has plainly been attended with a large measure of success, it is clear that the civilians and the Regulars have pulled well together, and that the results of their intermingling have been on the whole harmonious. It is worth while, in weighing up the work of the Corps, to take note of the fusion, and to examine briefly the relations which have been established between the professional soldier and his civilian comrade. It would be unfair to compare the two classes as professional and amateur soldiers; for though the temporarily commissioned are in very many cases distinctly amateurish as soldiers, the mere facts that both types are on one footing as qualified medical practitioners, and that the civilian is often the better equipped professionally of the two, prevent any valid comparison such as might reasonably be drawn between the combatant officers of the old and new Armies.

For a long time past to the officers of the Regular Royal Army Medical Corps have more and more been assigned administrative functions. Even men who at the beginning of the war were not very senior captains are now majors with commands of their own; and practically every R.A.M.C. officer of fifteen years' standing is now a lieutenant-colonel, and holding some position of great administrative responsibility. Most of the medicine and surgery at the bases, as well as almost all that at the casualty clearing-stations and field-ambulances, is being done by temporary officers; while hardly any others are to be found in combatant units or doing the collecting duties in the bearer divisions of the field-ambulances. It thus comes about that the Regular Army medical officers are almost wholly in positions of command, and the civilians almost wholly in positions of subordination to them; and the tendency in this direction has been increasing steadily ever since the war began. Right at the commencement this was not so: the temporarily commissioned were in a minority, and very large numbers of the junior officers of the R.A.M.C. were in subordinate positions.

### Cordiality and Solidarity.

It stands to reason, therefore, that any antagonism between the one class and the other would lead to friction and to diminution of efficiency. The almost unanimous testimony of those civilian doctors who return from the Front on leave or on expiration of their periods of service, is that both on and off duty they experience the greatest consideration and kindness from their commanding officers of the Regular Corps, and that the most cordial relations exist in general between the professional and the temporary members of the Medical Service. With this general statement few, one feels sure, will disagree; and it is very reassuring to hear on all sides such consensus of testimony to that effect. The mere fact that all are members of the same profession may have something to do with the solidarity of the R.A.M.C. abroad; be that as it may, its existence is a matter for congratulation to all concerned.

As a general rule, to which there are occasional exceptions, however, barrack-square formalities are cut down to the minimum in the Expeditionary Force; and in no branch is this so true as in the medical branch of it. Useless parades of all sorts are discouraged in nearly every unit, and strong common sense is exercised by most commanding officers in the matter of roll calls and similar routine. Saluting, of course, is insisted on both for men and officers; and when a parade of any kind is ordered, those who are warned for it must attend punctually and discharge their duties properly. But as a rule only those whose attendance is really required are warned, and only such parades ordered as are necessary for discipline and for the proper working of the unit. There are, or have been, a certain number of exceptions to this. One has heard of medical units in which four parades a day, for no purpose but that of keeping officers and men "on the go," have been the regular programme, and orderly officers are kept on the trot by pipe-clay ordinances from dawn till dark; but these are the exception, and those commanding officers who haze their men and officers thus are nowhere so severely criticised as by their own brother officers in the Regular R.A.M.C. Only lately one has heard of a small hospital unit with five junior officers available for orderly duty, in which a commander of this type deems it necessary to have two of them on orderly duty at a time, so that each of the medical officers is confined all day to camp, with practically nothing to do, for two days out of five.

"Militarism."

Similarly, in off-duty hours in nineteen cases out of twenty the R.A.M.C. commanding officers recognise in their civilian subordinates men like unto themselves, and treat them on their merits as colleagues and companions. A colonel will not disdain to play chess or picquet with a subaltern, and all in the mess are on terms of friendliness, even in many cases of affection. But in rare cases, one hears, there are officers who do not think it consonant with their dignity, or their self-importance, to adopt this attitude. They never address their junior officers except by the stiff title of Captain This or Mr. That; nor may the latter address the former save in terms as precise. They object to any officer speaking to them whilst smoking a cigarette—off parade, be it understood, for no one would dream of doing so on parade—and they would not for worlds unbend sufficiently to take a hand at bridge or any other form of evening relaxation. Let it be emphasised again, though chapter and verse could be given for all these statements, that these are the occasional exceptions, and that "swank" of this kind is quite rare, and is condemned by none more than the general run of colonels and majors of the Regular Service.

There is one point on which, to be quite frank and accurate, occasional grumbling is heard amongst the practitioner-soldiers; and that is in regard to the distributions of "mentions in dispatches" and more tangible rewards for good service, such as decorations. As has been hinted, far and away the major part of the dangerous work in the field is done now by civilian soldiers; and most of the arduous professional work everywhere. But the greater part of these rewards for service go to the Regular officers, not to the temporary ones who so vastly outnumber them. It is, of course, only another phase of the old quarrel between the Staff and the regimental officers in the combatant arms of the Service: those who do most of the work and endure most of the danger get least of the recognitions. In a sense, the award of a decoration to a senior officer often implies merit not so much on his part as on that of those under his command; and in this sense certainly the temporarily commissioned have been highly appreciated. The fact remains, however, that the professional soldier gets more, and the civilian less, than his share of decorations, and that the disproportion is very marked indeed. Another point of view of this question is that such rewards make little or no difference in the future prospects of the civilian temporarily turned soldier; whereas in the case of the Regular officer in the R.A.M.C. they may affect the whole of his future career. One would not ask that fewer honours should fall to the professional soldier (of the R.A.M.C.), but only that more should be allotted to the temporarily enlisted, the Special Reserve, and the Territorial.

Medical Officers in Combatant Units.

Lastly, a word about the relations between the medical officers attached to combatant units and the members of the messes to which they are attached. Here the same general statement can be made with confidence, that the medical officers are treated with the greatest possible courtesy and kindness by their combatant brethren, and especially by the officers commanding. Broadly speaking, the medical officer stands or falls on his merits as a sportsman; if ever such a one finds himself not very popular among the mess the fault is almost certainly his own. Just at first—say, during the first three or four months of the campaign—there were a small number of commanding officers who thought it *infra dignitatem* for any but a Regular R.A.M.C. officer to be attached to their units; but that feeling did not survive very long and has completely vanished now.

Socially, as well as in military matters, those who think of taking commissions in the R.A.M.C., whether Territorial, Reserve, or temporary, can rest assured that their reception will be kindness itself and their welcome a warm one; in the genial atmosphere of the R.A.M.C. the man who cannot make friends must be of peculiar disposition. If some officers commanding tend towards petty and irritating restrictions they are few and far between; and any who happen to meet them can console themselves with the reflection that colonels and lieutenant-colonels seldom stay more than a few months in the same billet, and that the next "O.C." will almost certainly be of a different mould.

What of the views of the Regulars about the temporary officers? The lack of familiarity of the latter (or of some of them, let us say) with military etiquette and with military customs causes a good deal of quiet amusement; and the differences between the adaptability of different civilians to the military environment are often commented on. But in the main the Regulars get on as well with their temporarily commissioned colleagues as the latter do with the former. They have functions to perform which are but partly medical; and they rely very largely on their new and usually junior colleagues from civil practice for wound treatment and other strictly professional work. They are, many of them, quite qualified to take a hand in such work and keenly interested in it, which means that their advisory and supervisory part is all the better played. The principal result of the intermixture of the professional soldier and practitioner in this war is a great increase of their mutual esteem.

